# Chronic kidney disease entertained from Lindsay's nails: A case report and literature review

**DOI:** 10.1002/ccr3.4426

**Published:** 2021-07-06

**Authors:** Saud Mohammed Raja

**Affiliations:** ^1^ Department of Internal Medicine Orotta National Referral Hospital/Orotta School of Medicine and Dentistry Asmara Eritrea

**Keywords:** case report, chronic kidney disease, half‐and‐half nails, Lindsay's nails

## Abstract

The presence of Lindsay's nails in a patient with a risk factor for chronic kidney disease could be a valuable clue to an underlying long‐standing renal impairment, particularly when serum creatinine level is not at hand.

## Graphical abstract

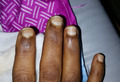

## 1 INTRODUCTION

Renal impairment is commonly diagnosed from raised blood levels of urea and creatinine. As chronic kidney disease is usually silent, delays in diagnosis are not uncommon. Thus, a higher degree of suspicion is indispensable. Detection of Lindsay's nails in this hypertensive patient was a significant hint for chronic kidney disease.

Chronic kidney disease (CKD) usually presents with persistently elevated serum urea and creatinine levels. However, due to the silent nature of CKD, the diagnosis can frequently be delayed if a timely renal function test is not carried out. Unfortunately, biochemical tests for the assessment of renal function may not be done in a patient for various reasons. Firstly, asymptomatic CKD patients tend not to seek medical help. Secondly, the test may not be requested in the first visit unless the patient is on a regular follow‐up for certain medical conditions. Thirdly, notably in resource‐limited settings, access to automated laboratory tests could be lacking in reasonable proximity. Hence, a higher degree of suspicion for renal impairment especially in those with risk factors is crucial. Utilizing all available clinical clues can also narrow down the differential diagnoses.

Advanced CKD presents with uremia. Although uremia consists of myriad symptoms and signs that demonstrate renal insufficiency, most of them are nonspecific to kidney disease. Nail changes are one of these non‐specific signs. Nail disorders commonly associated with chronic renal impairment include absent lunula, half‐and‐half nails, and splinter hemorrhages.^1^ Herein, I present a case of a 50‐year‐old woman with neglected CKD presenting for the first time to the emergency department with uremic symptoms. Recognition of Lindsay's nails in this patient despite lack of access to renal function test was an invaluable hint to the underlying renal impairment.

## 2 CASE REPORT

A 50‐year‐old woman with a medical history of poorly controlled hypertension and right nephrolithiasis presented to the emergency room with the complaint of a new‐onset generalized tonic‐clonic seizure. She also complained of headache, nausea, restlessness, headache, light‐headedness, fatigue, and shortness of breath on exertion. The patient denied any urinary complaint. She was not on outpatient follow‐up, and she quit antihypertensive medication for three months.

Her initial vitals were blood pressure 220/120 mm Hg, heart rate 113 beats per minute, respiratory rate 37 breaths per minute, and oxygen saturation 94% on room air. She was agitated but was alert and oriented. Her cardiopulmonary examination was notable for sinus tachycardia and tachypnea with normal breath sounds bilaterally.

As the first episode of seizure occurred at home, emergency management focused on controlling blood pressure, which was initially assumed to be the probable cause of the convulsion. Sublingual drops of nifedipine resulted in minimal blood pressure decrease. Four hours later, the patient had an episode of generalized tonic‐clonic convulsion that was aborted at the second minute using diazepam 10 mg given intravenously after securing the airway. The subsequent blood pressure record was 203/111 mm Hg. Hydralazine 10 mg was slowly administered intravenously. Although no other episode of convulsion occurred, blood pressure was not adequately controlled with those available medications.

**FIGURE 1:**
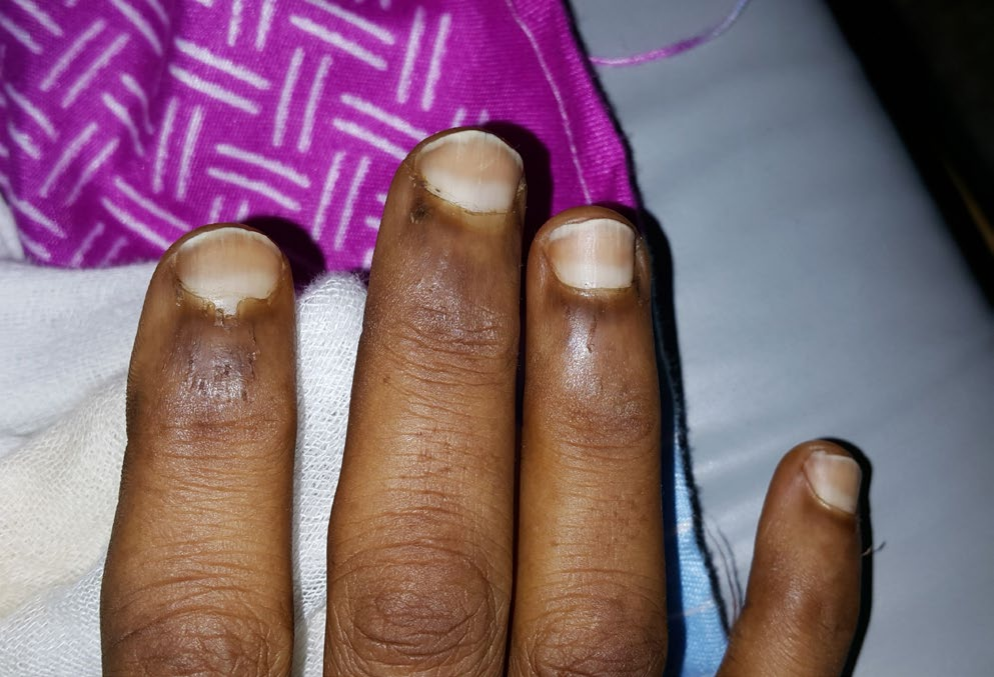
Fingernails of the presented patient showing typical Lindsay's nails

Meanwhile, some nail color changes were incidentally noted during a meticulous assessment of the patient. The fingernails had a whitish proximal half with dark‐brown sharply demarcated distal half that does not fade on pressure, fitting the appearance of Lindsay's nails (Half‐and‐half nails) (Figure 1). Taking the risk factors for CKD and the refractory hypertension into account, end‐stage renal disease (ESRD) was entertained. Unfortunately, renal function test was not immediately available in the setting.

When renal function test became accessible during working hours, blood urea nitrogen (BUN) was found to be 183 mg/dL and serum creatinine 17.2 mg/dL. Complete blood count showed hemoglobin of 7.2 g/dL. Ultrasound of the kidneys revealed shrunken kidneys with loss of cortico‐medullary differentiation. The patient was soon referred for dialysis with the provisional diagnosis of ESRD secondary to hypertensive nephropathy. Being into hemodialysis within 16 hours of her presentation, the patient was clinically stabilized. Blood pressure was controlled, and the patient showed marked clinical improvement upon subsequent evaluation.

## 3 DISCUSSION

Nail changes are common in chronic kidney disease, affecting more than 75% of hemodialysis patients.^2^ The two most common nail disorders among ESRD patients on hemodialysis are absent lunula and Lindsay's nails.^3^, ^4^ Lindsay's nails, also known as half‐and‐half nails, were first reported by Bean^5^ in 1964 in azotemia and later described and coined "half‐and‐half nails" by Lindsay^6^ in 1967 in a series of 1500 patients with chronic kidney disease. Lindsay's nails are seen in 20%‐50% of patients with chronic kidney disease.^7^ The nail disorder is characterized by nails that show the proximal portion of the nails white and the distal half red, pink, or brown, with a sharp line of demarcation between the two halves.^2^ The discolorations do not disappear under pressure, and there is no tendency to change the configuration with the growth of the nail. The change may affect one or all nails of the fingers and toes.^8^ As depicted in the picture, the nail changes in the present patient precisely fit the above‐mentioned description.

The pathophysiology of half‐and‐half nails is not clearly elucidated. The proximal white band is thought to result from chronic anemia secondary to an increased wall thickness of capillaries or overgrowth of connective tissue between the nail and the bone with the reduction in blood in the subpapillary plexus. The distal brown band is caused by melanin deposits, possibly from increased β‐melanocyte‐stimulating hormone.^9^ It was proposed that uremic toxins not only stimulate nail matrix melanocytes to produce melanin, but also slow nail growth rate resulting in larger accumulation of the pigment.

Lindsay's nails are commonly associated, but not specific, with chronic kidney disease. There is no correlation between the presence of Lindsay's nails and the degree of renal impairment or blood urea nitrogen or creatinine levels. It is also found that it does not improve with hemodialysis, although the nail discoloration may regress after successful renal transplantation.^1^ Apart from chronic kidney disease, Lindsay's nails have also been reported in cirrhosis, Crohn's disease, Behçet's disease, pellagra citrullinemia, Kawasaki disease, and even in healthy persons.^8^ They are also reported in a patient with hepatocellular carcinoma.^10^

Lindsay's nails should not be confused with other nail disorders particularly with Terry's nails. In Terry's nails, the distal 1‐2 mm of the nail shows a pink or brown band with the entire nail plate or proximal end whiteness. These changes have been noted in 25% of hospitalized patients, most commonly those with cirrhosis, chronic congestive heart failure, and adult‐onset diabetes, and in very elderly patients.^2^

## 4 CONCLUSION

Lindsay's nails are relatively uncommon clinical examination findings and probably unheard‐of to many clinicians. With the advent of the rapid contemporary technological dependence in modern medicine, these signs might be considered obsolete and of no use. However, the present case report demonstrates the importance of subtle physical examination clues in the diagnosis of chronic kidney disease especially in the absence of relevant laboratory investigations. Although this patient with no prior follow‐up to a primary care physician presented with a complicated CKD, the emergency department is, in fact, not the ideal setting to look for these subtle signs.

The recognition of these associated nail disorders might help early consideration of possible renal insufficiency in a patient with known risk factors even in cases where there is no significant rise in serum urea or creatinine considering the lack of its correlation with those laboratory parameters. On top of that, since the nail changes are associated with chronic kidney disease instead of acute kidney injury, the presence of half‐and‐half nails in the background of confirmed renal insufficiency would also make acute kidney injury unlikely, thereby assisting in differentiating chronic from acute. Therefore, clinicians should be aware of this clinical sign and look for it in their daily practice when appropriate. Nevertheless, it is prudent to keep in mind that, like many clinical signs, its presence is more clinically relevant and helpful in decision‐making than the absence of thereof.

## CONFLICT OF INTEREST

None declared.

## AUTHOR CONTRIBUTIONS

Saud Mohammed Raja detected the clinical sign, took the lead in the management of the patient, did the photography, and wrote the manuscript.

## ETHICAL APPROVAL

Written informed consent was obtained from the patient for publication of this case report and the related photograph.

## Data Availability

Data sharing is not applicable to this article as no new data were created or analyzed in this study.

## References

[1] Neild GH , Alston H , Burns A . Half and half nails. Clin Kidney J. 2011;4(5):361. 10.1093/ndtplus/sfr087 PMC4421742 25984192

[2] James WD , Berger TG , Elston DM . Andrew's Diseases of the Skin. E‐Book. Elsevier Health Sciences; 2011:773 p.

[3] Martinez MA , Gregório CL , Santos VP , Bérgamo RR , Machado Filho CD . Nail disorders in patients with chronic renal failure undergoing hemodialysis. An Bras Dermatol. 2010;85(3):318‐323. 10.1590/s0365-05962010000300004 20676464

[4] Al‐Hamamy HR , Al‐Mashhadani SA , Fadheel BM , Salman SH . Nail changes in hemodialysis patients and renal transplant recipients (a case‐control study). Am J Dermatol Venereol. 2014;3(2):30‐34. 10.5923/j.ajdv.20140302.02

[5] Bean WB . Nail growth: a twenty‐year study. Arch Intern Med. 1963;111(4):476‐482. 10.1001/archinte.1963.03620280076012 13969933

[6] Lindsay PG . The half‐and‐half nail. Arch Intern Med. 1967;119:583‐587. 10.1001/archinte.1967.00290240105007 6027185

[7] Khanna D , Singal A , Kalra OP . Comparison of cutaneous manifestations in chronic kidney disease with or without dialysis. Postgrad Med J. 2010;86:641‐647. 10.1136/pgmj.2009.095745 21037238

[8] Oanță A , Iliescu V , Țărean S . Half and half nails in a healthy person. Acta Dermatovenerol Croat. 2017;25(4):303‐304. 30064606

[9] Leyden JJ , Wood MG . The “Half‐and‐Half nail”. Arch Dermatol. 1972;105:591‐592. 10.1001/archderm.1972.01620070063024 5017274

[10] Jamwal V , Chandail VS . Half and half nails: not always uremia. JK Sci. 2010;12(3):161.

